# Evolution of Pathology Patterns in Persons Who Died From COVID-19 in Italy: A National Study Based on Death Certificates

**DOI:** 10.3389/fmed.2021.645543

**Published:** 2021-03-22

**Authors:** Francesco Grippo, Enrico Grande, Alice Maraschini, Simone Navarra, Marilena Pappagallo, Stefano Marchetti, Roberta Crialesi, Luisa Frova, Chiara Orsi, Silvia Simeoni, Annamaria Carinci, Giuseppe Loreto, Chiara Donfrancesco, Cinzia Lo Noce, Luigi Palmieri, Xanthi Andrianou, Alberto Mateo Urdiales, Graziano Onder, Giada Minelli, Italian National Institute of Health COVID-19 Mortality Group

**Affiliations:** ^1^Division of Integrated Systems for Health, Social Assistance and Welfare, Italian National Institute of Statistics, Rome, Italy; ^2^Statistical Service, Istituto Superiore di Sanità, Rome, Italy; ^3^Department of Cardiovascular, Endocrine-metabolic Diseases and Ageing, Istituto Superiore di Sanità, Rome, Italy; ^4^Department of Infectious Diseases, Istituto Superiore di Sanità, Rome, Italy

**Keywords:** SARS-CoV-2, mortality, cause of death, comorbidities, surveillance

## Abstract

**Background:** In Italy, during the first epidemic wave of 2020, the peak of coronavirus disease 2019 (COVID-19) mortality was reached at the end of March. Afterward, a progressive reduction was observed until much lower figures were reached during the summer, resulting from the contained circulation of SARS-CoV-2. This study aimed to determine if and how the pathological patterns of the individuals deceased from COVID-19 changed during the phases of epidemic waves in terms of: (i) main cause of death, (ii) comorbidities, and (iii) complications related to death.

**Methods:** Death certificates of persons who died and tested positive for SARS-CoV-2, provided by the National Surveillance system, were coded according to ICD rev10. Deaths due to COVID-19 were defined as those in which COVID-19 was the underlying cause of death.

**Results:** The percentage of COVID-19 deaths varied over time. It decreased in the downward phase of the epidemic curve (76.6 vs. 88.7%). In February–April 2020, hypertensive heart disease was mentioned as a comorbidity in 18.5% of death certificates, followed by diabetes (15.9% of cases), ischemic heart disease (13.1%), and neoplasms (12.1%). In May–September, the most frequent comorbidity was neoplasms (17.3% of cases), followed by hypertensive heart disease (14.9%), diabetes (14.8%), and dementia/Alzheimer's disease (11.9%). The most mentioned complications in both periods were pneumonia and respiratory failure with a frequency far higher than any other condition (78.4% in February–April 2020 and 63.7% in May–September 2020).

**Discussion:** The age of patients dying from COVID-19 and their disease burden increased in the May–September 2020 period. A more serious disease burden was observed in this period, with a significantly higher frequency of chronic pathologies. Our study suggests better control of the virus' lethality in the second phase of the epidemic, when the health system was less burdened. Moreover, COVID-19 care protocols had been created in hospitals, and knowledge about the diagnosis and treatment of COVID-19 had improved, potentially leading to more accurate diagnosis and better treatment. All these factors may have improved survival in patients with COVID-19 and led to a shift in mortality to older, more vulnerable, and complex patients.

## Introduction

A key feature of the new pathogen SARS-CoV-2 is the causation of a severe disease (coronavirus disease 2019, COVID-19) characterized by a high rate of lethality. In Italy, the first ascertained COVID-19-related death was registered on February 21, 2020. Afterward, the number of deaths progressively increased, reaching a peak in March 2020 and then entering a descending phase until September 2020 with 35,457 total deaths, of which 84% occurred within May (1). From the beginning of the pandemic, Italy has been among the countries with the highest mortality from COVID-19 worldwide (2, 3).

As described elsewhere, (4) the national surveillance system managed by the Italian National Institute of Health registered all COVID-19 cases and collected death certificates of those who died, regardless of whether COVID-19 was the underlying or associated cause of death.

The first analysis of those death certificates, collected from the beginning of the pandemic until May 2020, pointed out that 88% of the recorded deaths had COVID-19 as the direct (underlying) cause of death, with slightly higher proportions among men and in the population aged 60–79 years (4).

Similar studies in other countries have reported that COVID-19 was a very significant cause of death in Europe during the first wave of the pandemic, e.g., in England, data from the Office for National Statistics showed that COVID-19 was to blame for one-quarter of all deaths in April 2020 (*n* = 33,841, 26.7% of the total deaths)^1^ (5), and the role of comorbidities was also explored in the UK data (6).

In Italy, the peak of mortality from COVID-19 was reached on March 28 with 925 deaths. Afterward, a progressive reduction of the number of deaths was observed (7), until much lower figures were reached during summer (average of 14 deaths per day), resulting from contained virus circulation.

The present study aimed to determine, through the analysis of death certificates, whether and how the pathology patterns of individuals deceased from COVID-19 have changed during the phases of the epidemics in terms of (i) the main cause of death, (ii) co-morbidities, and (iii) complications related to death.

## Materials and Methods

The COVID-19 surveillance system managed by the Italian National Institute of Health (Istituto Superiore di Sanità; ISS) collects information on all SARS-CoV-2-positive individuals throughout the country (1, 3). In this framework, regions and autonomous provinces are required to provide death certificates of SARS-CoV-2-positive people. A joint group of researchers from the ISS and Italian National Institute of Statistics (Istat) was established to analyze these certificates.

This paper describes a comparison of the results of cause of death analysis during two different periods of the pandemic: February–April 2020, when the epidemic had a high impact on the Italian population, and May–September 2020, characterized by less effective viral circulation and reduced COVID-19 mortality.

Between February 21 and September 30, 2020, 35,457 deaths in SARS-CoV-2-positive patients were reported in Italy. Of that total, 35,440 were at least 30 years old, We focused on this age group since in the younger people the mortality is often due to other preexisting conditions.

**Figure 1:**
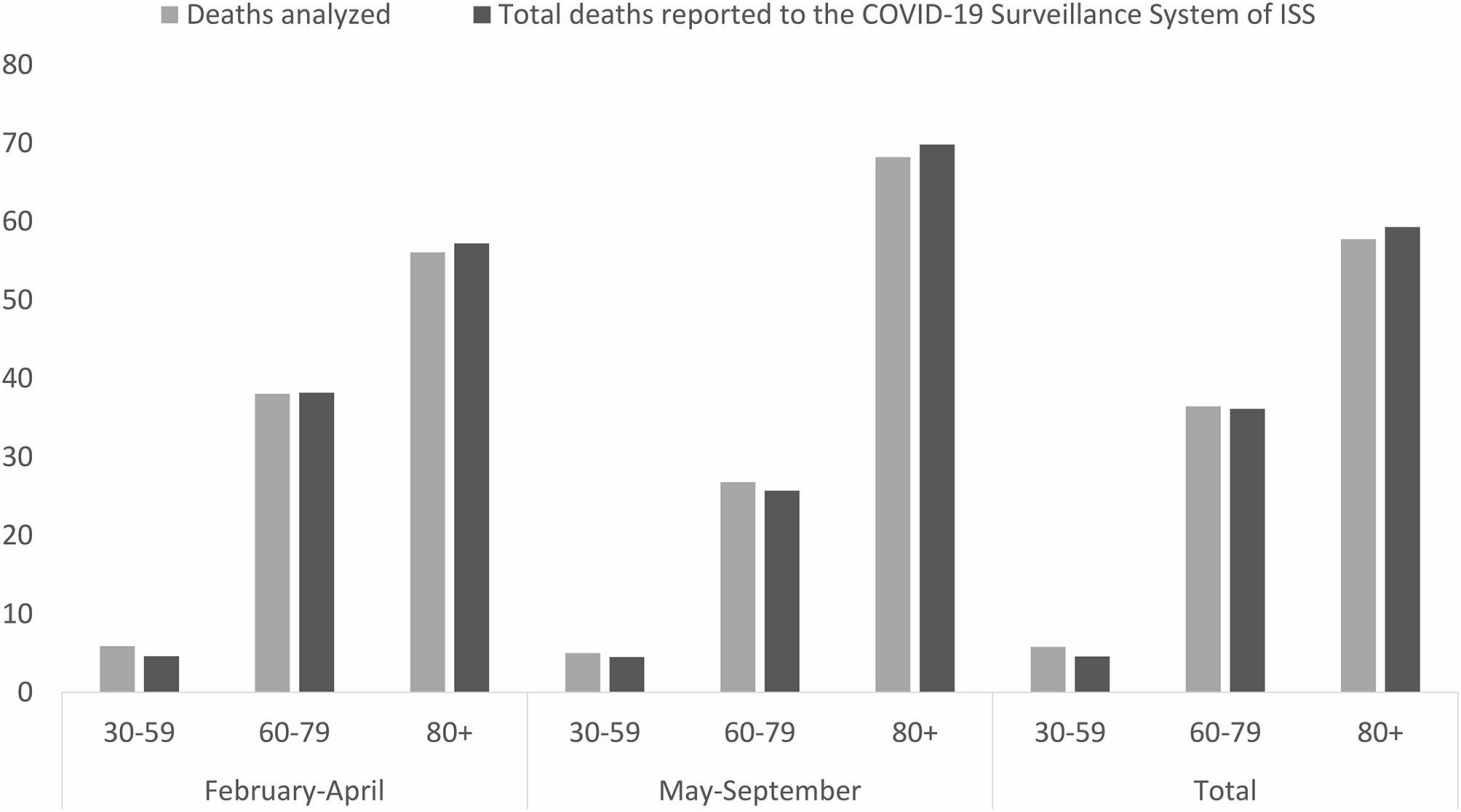
Age distribution of analyzed deaths and deaths reported to the COVID-19 Surveillance System of ISS in the periods February–April and May–September 2020.

The present analysis considered a sample of 5,662 death certificates corresponding to 16% of the above-mentioned 35,440 deaths occurring in the study periods. The sample selection is based of demographic and geographical distribution, trying to preserve a proportionality with respect to the total number of deaths. Death certificates had the following age distribution: 30–59 years: 287 in February–April and 40 May–September; 60–79: 1.850 and 214; 80 years and older 2.726 and 545. Age and sex distribution were similar to that of all COVID-19 deaths in both analyzed periods (Figure 1), and they were distributed all over the country.

The causes of death reported on death certificates were classified by Istat according to the International Classification of Diseases (ICD10) (8). For each death certificate, the underlying cause of death was identified, in line with the WHO definition, as “the condition initiating the train of morbid events directly leading to death.” ICD10 coding was performed using the worldwide-used software Iris^2^ and software's rejects were reviewed by expert coders.

All reported causes were then categorized according to their role in the death process as either of the following:

- comorbidities: conditions “reported in the certificate different from” COVID-19 and “not caused by it.” A pre-existing validated algorithm, developed for the study of multiple causes of death, was used to select comorbidities.
- complications of COVID-19: conditions reported by certifiers as “originating from” COVID-19.

The methodology used for selecting comorbidities and complications of COVID-19 was extensively described elsewhere (9). Table 1 lists the analyzed conditions and the respective ICD10 codes.

**Table 1:** Comorbidities and complications of COVID-19 analyzed with ICD10 codes.

**Comorbidities**	**ICD10 codes**	**Conditions reported as complications of COVID-19**	**ICD10 codes**
Infectious and parasitic diseases	A00–B99	Sepsis, septic shock, and infections	A40–A41, A49, B25–B49, B99, R572
Neoplasms	C00–D48	Dehydration	E86
Diabetes	E10–E14	Encephalitis	G04, G93
Obesity	E66	Acute myocardial infarction	I21
Dementia and Alzheimer's	F01–F03, G30	Pulmonary embolism	I26
Hypertensive heart diseases	I10–I15	Heart complications	I50–I51
Ischemic heart disease	I20–I25	Cerebrovascular accidents	I60–I64
Cerebrovascular diseases	I60–I69	Respiratory distress and pulmonary edema	J80–J81
Other respiratory diseases	J00–J99	Intestinal complications	A00–A09, K50–K67
Other diseases of the circulatory system	I00–I09, I30–I51, I70–I99	Renal failure	N17, N19
Chronic lower respiratory diseases	J40–J47	Shock (cardiogenic)	R57 (excluding R572)
Chronic liver diseases	K70–KB		
Renal failure	N17–N19		
External causes	S00–T98		

Absolute and percent frequencies of certificates with Covid-19 as underlying cause, comorbidities and complications as well as the average number of comorbidities reported were computed. Logistic regression models were applied to identify which comorbidities and complications are mostly associated with the period of death (used as independent variable of the model). A separate age and sex adjusted model was performed for each comorbidity or complication. Odds ratios (ORs) with 95% confidence intervals (CIs) were computed using the period February-April as reference.

### Ethical Issues

On February 27, 2020, the Italian Presidency of the Council of Ministers in compliance with the European General Data Protection Regulation (UE GDPR 2016/679) authorized the processing of personal data related to COVID-19 by the ISS and other public institutions for reasons of public interest in public health^3^.

## Results

Of the 5,662 analyzed death certificates, 3,447 (60.9%) were for men and 2,215 (39.1%) for women; 327 (5.8%) deaths occurred in ages 30–59 years, 2,064 (36.4%) in ages 60–79 years, and 3,271 (57.8%) in ages 80 years or older. Most analyzed deaths (4,863 or 85.9% of the total) occurred in February–April 2020 (only 37 deaths occurred in February). In this period, males accounted for 63% of the total, whereas the percentage of males in May–September 2020 dropped to 48%. The age distribution was also slightly different in the two periods: average age was 79.2 (±0.1) and 81.9 (±0.4) in the first and second periods, respectively. Deceased who aged 80 years or older increased from 56% (the first period) to ~70% (the second period) (Figure 1).

Table 2 shows some descriptive indicators concerning cause of death analysis. Overall, COVID-19 was the underlying cause of death in 87.2% of all deaths with differences in the two periods: 88.7 and 76.6% in the first and second periods, respectively.

**Table 2:** Descriptive indicators of causes of death reported on death certificates.

	**February–April**	**May–September**	**Total**
Number of deaths analyzed	4,863	799	5,662
COVID-19 underlying cause of death (percentage of death certificates)	88.7	76.6	87.2
Non-COVID-19 underlying cause of death (percentage of death certificates)	11.3	23.4	12.8
Average number of comorbidities (± standard error)	1.28 ± 0.03	1.52 ± 0.07	1.31 ± 0.03
Certificates with comorbidities besides COVID19 (percentage)	71.6	81.6	73.0

The average number of comorbidities reported on death certificates was 1.28 and 1.52 in the first and second periods, and the percent of cases with comorbidities listed among causes of death increased from 71.6 to 81.6%, respectively.

### Analysis of Comorbidities

**Figure 2:**
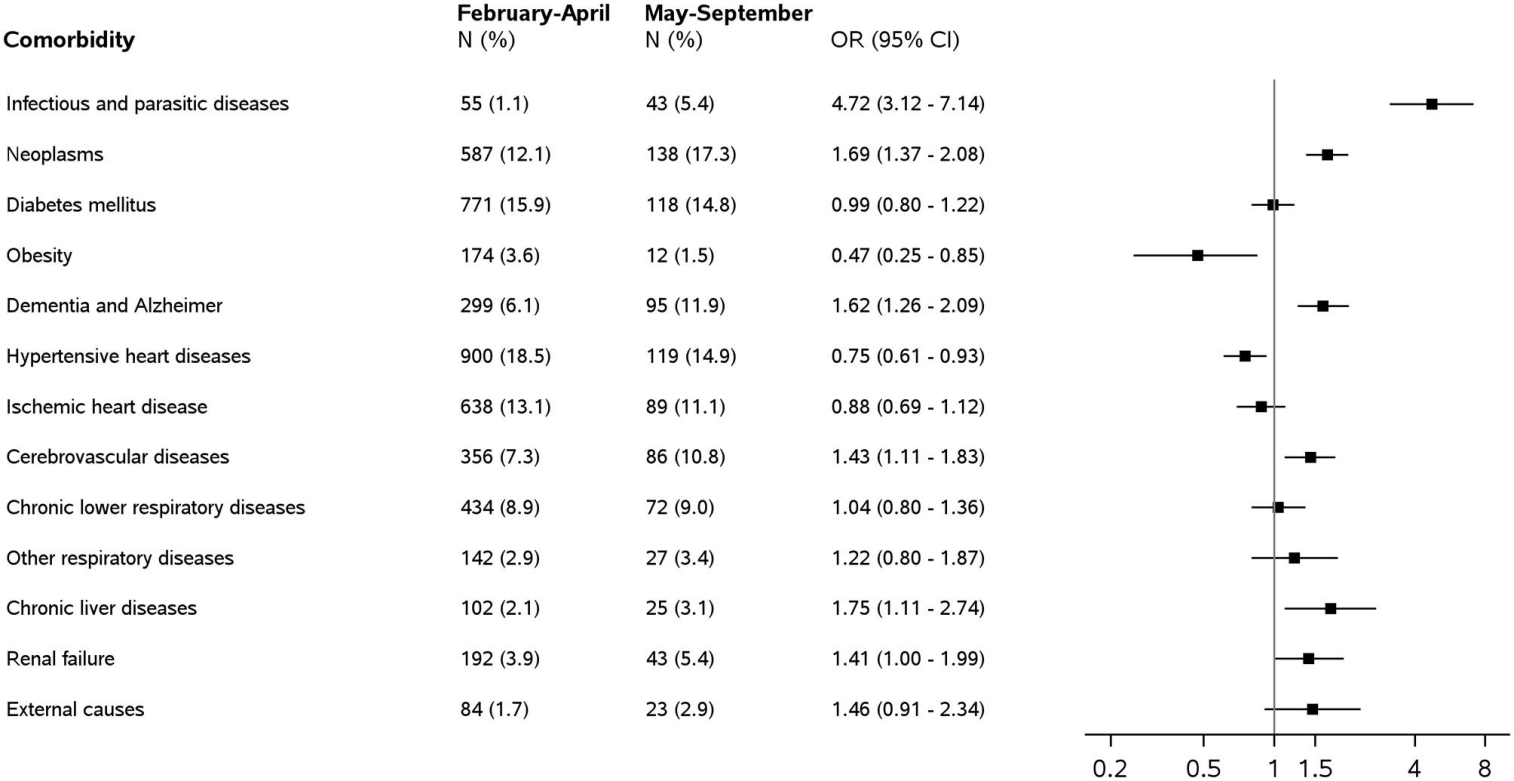
Comorbidities on death certificates of people who tested positive for Sars-COV-2 by type and period: frequency (*N*), percentage of the total number of death certificates (%), and odds ratios (OR) with 95% confidence intervals (CIs) for the association between comorbidities and deaths occurring in May–September 2020 compared with those in February–April 2020. Analyses are adjusted by age and gender.

Figure 2 shows the percentage of certificates reporting each comorbidity for the two periods, together with age and sex-adjusted ORs of the risk of being reported in May–September 2020 compared with those reported in February–April 2020.

The average number of comorbidities reported was 1.28 (±0.03 standard error) in the first period and 1.52 (±0.07) in the second period.

Neoplasms, hypertensive heart diseases, and diabetes were among the most frequently mentioned comorbidities with significant differences in the two periods. In February–April 2020, hypertensive heart disease was mentioned in 18.5% of death certificates, followed by diabetes (15.9% of cases), ischemic heart . disease (13.1%), and neoplasms (12.1%). In May–September 2020, the most frequent comorbidity was neoplasms (17.3% of cases), followed by hypertensive heart disease (14.9%), diabetes (14.8%), and dementia/Alzheimer's disease (11.9%).

Also, age and sex-adjusted ORs showed that hypertensive heart diseases and obesity were significantly less frequently reported in May–September 2020 than in February–April 2020. OR was 0.75 (95% CI 0.61–0.93) for hypertensive heart diseases and 0.47 (95% CI 0.25–0.85) for obesity.

Comorbidities reported more frequently in May–September 2020 were neoplasms (OR = 1.69, 95% CI 1.37–2.08), dementia/Alzheimer's disease (OR = 1.62, 95% CI 1.26–2.09), cerebrovascular diseases (OR = 1.43, 95% CI 1.11–1.83), infectious and parasitic diseases (OR = 4.72, 95% CI 3.12–7.14), and chronic liver diseases (1.75, 95% CI 0.80–1.36).

As comorbidities more frequently observed in the second period seemed to be related to the older age of the decedents, ORs were estimated for the age stratum of 80 years and over, but no differences from the results obtained with the non-stratified models were observed.

### Analysis of Complications

**Figure 3:**
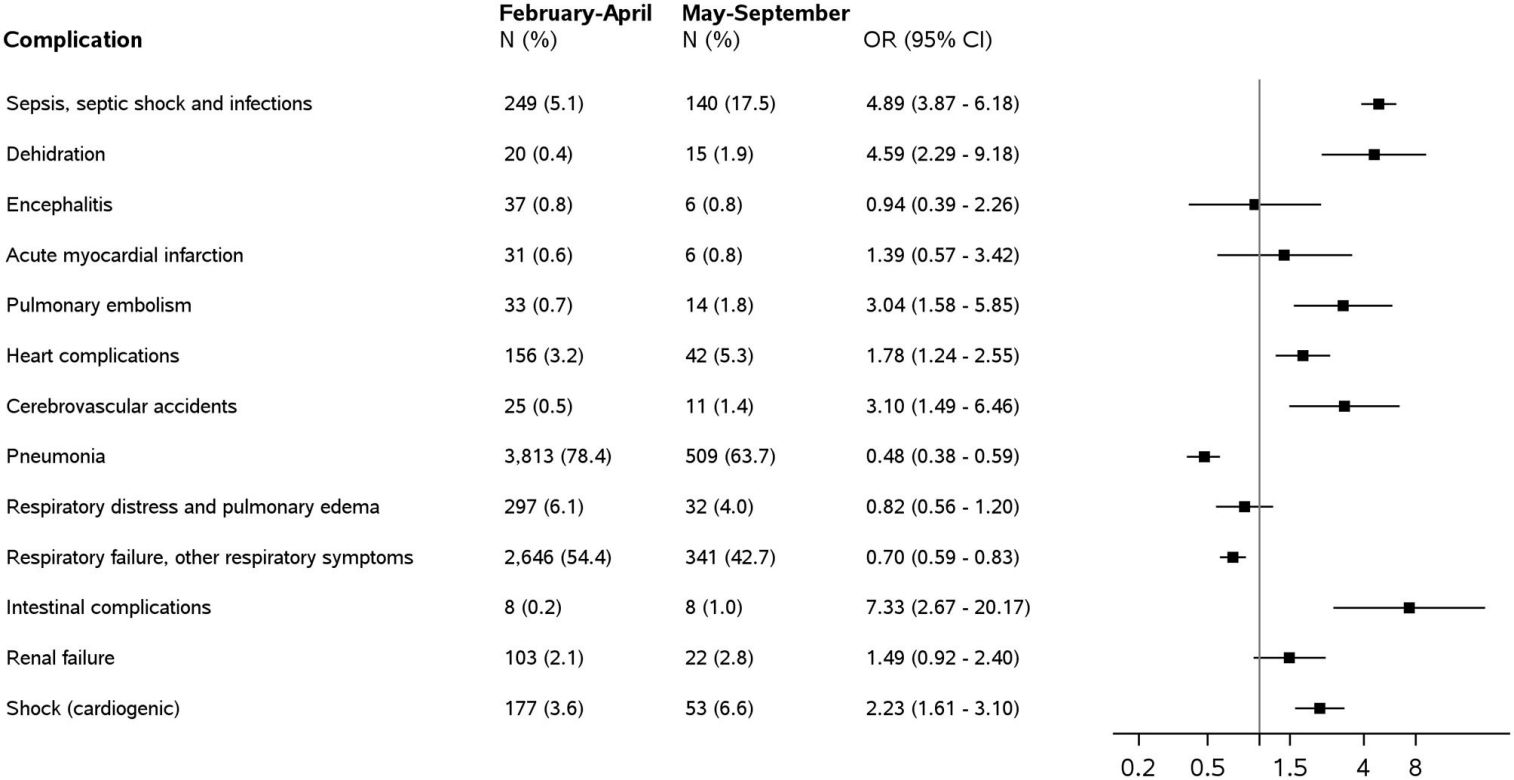
Conditions reported as complications of COVID-19 on death certificates of people who tested positive for Sars-COV-2 by type and period: frequency (*N*), percentage of the total number of death certificates (%), and odds ratios (ORs) with 95% confidence intervals (CIs) for the association between complications and deaths occurring in May–September 2020 compared with those in February–April 2020.

Figure 3 shows the percentage of each condition reported as a complication of COVID-19 together with age and sex-adjusted ORs for the association between complications and deaths occurring May–September 2020 compared with those in February–April 2020.

The most mentioned complications were pneumonia and respiratory failure in both periods with frequencies far higher than any other conditions.

Pneumonia was reported as a complication of COVID-19 in 78.4% of deaths in February–April 2020 and 63.7% in May–September 2020, with respiratory failure in 54.4 and 42.7% of cases, respectively. Moreover, these respiratory conditions are the only complications showing higher frequencies in the first period.

Among others, the following complications were found more frequently in May–September 2020: sepsis and infections unspecified (OR = 4.89, 95% CI 3.87–6.18), heart complications (OR = 1.78, 95% CI = 1.24–2.55), and pulmonary embolism (OR = 3.04, 95% CI = 1.58–5.85).

## Discussion

Excess mortality due to COVID-19 during the peak of the first epidemic period has been widely reported in the literature (10–14), whereas studies on the individual causes of death are scarce and based on small series. As reported by WHO, “death is defined for surveillance purposes as a death resulting from a clinically compatible illness in a probable or confirmed COVID-19 case”; however, this definition could lead to different interpretations. In fact, most countries have different approaches to determining the exact numbers of COVID-19 deaths, and few systems can produce cause of death statistics based on the underlying cause criteria in ICD10.

Our analysis performed on 5,662 death certificates has shown that among patients positive for SARS-CoV who died, the percentage of deaths presenting COVID-19 as the underlying cause varied over time. Particularly, it decreased in the downward phase of the epidemic curve (76.6 vs. 88.7%). Additionally, the age of patients dying with COVID-19 and their disease burden increased in the second epidemic period from May to September 2020. A more serious disease burden was observed in this period, with a significantly higher frequency of chronic pathologies such as dementia and Alzheimer's disease (15, 16), cerebrovascular diseases (17), diseases of the blood and hematopoietic system, diseases of the digestive system (18), and chronic liver diseases (19, 20).

These data suggest improved control of virus lethality or at least its mitigation in less fragile groups of the population and could be explained by different factors. First, there was less burden on the healthcare system in the second period of the epidemic. In the peak of the first period, emergency rooms, hospitals, and intensive care units were challenged by the need to simultaneously provide care to a high number of critically ill patients. Second, the organization of care improved in the second period of the epidemic. COVID-19 and non-COVID-19 care protocols and workflows were created in hospitals, community care approaches were developed, and specific diagnostic and therapeutic processes were implemented. Finally, knowledge of COVID-19 diagnosis and treatment improved over time, potentially leading to more accurate diagnosis and better treatment. All these factors may have improved survival in patients with COVID-19 and led to a shift of mortality toward older, more vulnerable, and complex patients (21).

The presence of some of these pathologies has already been dealt with in the literature, regarding COVID-19. The proportion of deaths without any contributing cause has decreased. Therefore, the reduced stress upon the national health system clearly seems to have played a major role in mitigating the impact of the pandemic. A separate focus should be put on infectious and parasitic diseases, which presented high odds in the second observational period. Such evidence is difficult to explain, although it may be associated with a more general organic decay in patients with severe forms of COVID-19, leading to a greater predisposition to develop infections.

Another relevant feature seems to regard the overall complications documented during the second period: during the epidemic peak, pneumonia and respiratory failure were the most relevant complications (4). These complications were significantly reduced when the outbreak was under control, thanks to prevention and mitigation progress (19). Moreover, the complications mentioned on death certificates collected during the second period were characterized by a high prevalence of sepsis, septic shock and infections, dehydration, and intestinal complications. These complications could be suggestive of a more systemic perspective of severity. Additionally, we can hypothesize that in the second epidemic period, typical COVID-19 respiratory conditions were better treated and managed, so death may have occurred when patients experienced additional non-respiratory complications that further worsened health status, leading to a negative prognosis.

A possible limitation of the present study relates to the generalizability of our findings to other countries. Italy has the oldest population in Europe and given the impact of age on the development of chronic conditions (comorbidities) it might be hypothesized that their occurrence in persons dying with COVID-19 might be higher than in other countries with a younger population. Also the older age of the Italian population can give reason to the higher COVID-19 mortality rate observed in Italy as compared with other countries. In addition, the organization of the health care systems (including the availability of hospital and intensive care unit beds) and its responsiveness to the epidemic might vary from other countries and this might explain differences among countries in mortality rate and in characteristics of persons dying with COVID-19.

Finally, the comparison between mortality observed during the ascending and descending phases of the epidemic curve has allowed us to confirm what was already observed. Mortality was strongly connected to SARS-CoV-2 circulation and, consequently, to a different pressure on the national health service.

## Data Availability Statement

The raw data supporting the conclusions of this article will be made available by the authors, without undue reservation.

## Ethics Statement

The studies involving human participants were reviewed and approved by on February 27, 2020, the Italian Presidency of the Council of Ministers in compliance with the European General Data Protection Regulation (UE GDPR 2016/679) authorized the processing of personal data related to COVID-19 by the ISS and other public institutions for reasons of public interest in public health. Written informed consent for participation was not required for this study in accordance with the national legislation and the institutional requirements.

## Author Contributions

FG, EG, and AM: contributed to the design of the study, performed the statistical analyses, and drafted the manuscript. SN, MP, SM, RC, LF, CO, and SS: contributed to the design of the study, and to the coding of mortality data. AC, GL, CLN, LP, and CD: contributed to the collection and management of mortality data. XA and AU: elaborated surveillance data. GO and GM: contributed to the conception and design of the study and revised the advanced draft of the manuscript. All authors approved the final manuscript as submitted and agree to be accountable for all aspects of the work.

## Conflict of Interest

The authors declare that the research was conducted in the absence of any commercial or financial relationships that could be construed as a potential conflict of interest.

## Footnotes

^1^Available online at: https://www.ons.gov.uk/peoplepopulationandcommunity/birthsdeathsandmarriages/deaths/bulletins/deathsinvolvingcovid19englandandwales/deathsoccurringinapril2020 (accessed February 18, 2020).

^2^Available online at: https://www.iris-institute.org (accessed February 18, 2020).

^3^Available online at: https://www.gazzettaufficiale.it/eli/id/2020/02/28/20A01348/SG (accessed December 21, 2020)
